# Molecular Epidemiology and Clinical Features of Enteroviruses-Associated Hand, Foot, and Mouth Disease and Herpangina Outbreak in Zunyi, China, 2019

**DOI:** 10.3389/fmed.2021.656699

**Published:** 2021-04-26

**Authors:** Yuanhang Ai, Weiwei Zhang, Jie Wu, Jingzhi Zhang, Meijing Shen, Shifei Yao, Chengmin Deng, Xiaoqian Li, Dejing Wu, Peng Tian, Xiaoju Cheng, He Zha, Kaifeng Wu

**Affiliations:** ^1^Department of Clinical Laboratory, Zunyi Medical University Third Affiliated Hospital, Zunyi, China; ^2^Department of Pediatrics and Child Health, Zunyi Medical University Third Affiliated Hospital, Zunyi, China; ^3^Department of Scientific Research Laboratory, Zunyi Medical University Third Affiliated Hospital, Zunyi, China

**Keywords:** molecular epidemiology, clinical features, enterovirus types, herpangina, hand foot and mouth disease

## Abstract

**Background:** Hand, foot and mouth disease (HFMD) and herpangina (HA), two of the most common childhood infectious diseases, are associated with enteroviruses (EVs) infection. The aim of this study was to identify the molecular epidemiology of enterovirus causing HFMD/HA in Zunyi, China, during 2019, and to describe the clinical features of the cases.

**Methods:** We collected the information on demographic and clinical characteristics, laboratory data of laboratory-confirmed EVs associated HFMD/HA cases in Zunyi Medical University Third Affiliated Hospital between March 1 and July 31, 2019. EV types were determined by either one-step real time RT-PCR or partial VP1 gene sequencing and sequence alignment. Phylogenetic analysis of CVA6, CVA2, and CVA5 were established based on the partial VP1 gene sequences by neighbor-joining method. Differences in clinical characteristics and laboratory results of the cases were compared among patients infected with the most prevalent EV types.

**Results:** From 1 March to 31 July 2019, 1,377 EVs associated HFMD/HA inpatients were confirmed. Of them, 4 (0.3%, 4/1,377) were EV-A71-associated cases, 84 (6.1%, 84/1,377) were CVA16-associated cases, and 1,289 (93.6%, 1,289/1,377) were non-EV-A71/CVA16-associated cases. Of the randomly selected 372 non-EV-A71/CVA16 cases, EV types have been successfully determined in 273 cases including 166 HFMD and 107 HA cases. For HFMD cases, the three most common types were CVA6 (80.7%, 134/166), CVA2 (5.4%, 9/166) and CVA5 (3.0%, 5/166); similarly, for HA cases, the three most prevalent serotypes were CVA6 (36.5%, 39/107), CVA2 (21.5%, 23/107) and CVA5 (18.7%, 20/107). Phylogenetic analysis showed that subclade D of CVA5, and subclade E of CVA6 and CVA2 were predominant in Zunyi during the outbreak in 2019. Compared with the cases caused by CVA16, the incidence of high fever and severe infection associated with CVA2, CVA5, and CVA6 was higher.

**Conclusions:** The recent HFMD/HA outbreak in Zunyi is due to a larger incidence of CVA6, CVA2, and CVA5. Novel diagnostic reagents and vaccines against these types would be important to monitor and control EV infections.

## Introduction

Hand, foot, and mouth disease (HFMD) and herpangina (HA) are the two major childhood infectious diseases caused by enteroviruses. HFMD/HA are recognized as self-limiting diseases, but may present with encephalitis, meningitis, myocarditis, acute flaccid paralysis, pulmonary edema in severe cases and can occasionally lead to death (1–4). HFMD/HA outbreaks occurred frequently worldwide, especially in the Asia–Pacific region. According to the most recent data released by Chinese Center for Disease Control and Prevention, ~2 million cases of HFMD were diagnosed in China, 2019, and 20 deaths were reported [quoted from (5)].

EVs belong to the family Picornaviridae, which comprises four human disease-associated species of Enterovirus A-D (6, 7). It has been reported that more than 20 EV types were capable to cause HFMD/HA (8). Of the EV types, enterovirus A71 (EV-A71) and coxsackievirus A16 (CVA16) has been recognized as the most predominant EV types associated with HFMD (9); while CVA2, CVA6, and CVA10 were the most prevalent types associated with HA (10, 11). Based on currently available data, it seems that CVA6 has replaced EV-A71 and CVA16 and became the most prevalent serotype associated with HFMD in regions of China (12–14). However, national epidemiological data on HFMD/HA associated EV types of recent 3 years remain scarce in China. We have observed that, between the year of 2012 and 2014, HFMD outbreaks were associated with EV other than EV-A71/CVA16, yet the subtype was not identified (15). Our limited knowledge on the epidemiology of EVs has hampered the development of effective diagnosis and prevention options. Therefore, the present study was conducted to illustrate the latest molecular epidemiology of enteroviruses causing HFMD/HA in Zunyi, China, and to compare the clinical characteristics between patients infected with the most common EV types.

## Methods

### Case Definitions

We collected and reviewed the electronic records of all cases with laboratory-confirmed HFMD/HA in Zunyi Medical University Third Affiliated Hospital (The First People's Hospital of Zunyi) between March 1 and July 31, 2019. Patients who presenting with oral vesicular exanthema/ulcers on the anterior tonsillar pillars, soft palate, buccal mucosa, or uvula are preliminarily diagnosed as clinical cases of HA, and patients who presenting with oral vesicular exanthema/ulcers on the tongue or the buccal mucosa, and vesicular rashes over the palms, soles, buttocks or the trunk are preliminarily diagnosed as clinical cases of HFMD. Laboratory-confirmed cases of HFMD/HA were diagnosed if a clinical case was tested positive for enterovirus nucleic acids. Patients were classified as severe in case they had any neurological complications, or cardiopulmonary complications (16, 17).

### Specimen, Data Collection, and Processing

Pharyngeal swabs were the only sample sources, and the aliquot of remaining clinical samples were stored at −80°C. The presence of enteroviruses and the types of EV-A71 and CVA16 were detected using real-time RT-PCR kits following the manufacturer's instructions (Da AN Gene Co. Ltd, Guangzhou, China). For identification of non-EV71/CVA16 types, 372 confirmed inpatient cases (216 HFMD cases and 156 HA cases) were randomly selected for further partial VP1 gene amplification and sequencing. The demographic information, clinical data, and laboratory findings of patients were retrospectively collected through the hospital health/laboratory information system.

### Partial VP1 Gene Amplification and Enterovirus Typing

Serotypes other than EV-A71/CVA16 were identified with reverse transcription-nested PCR (nested RT-PCR) as previously described (18). Viral RNA was extracted from 140 μl clinical samples using the Viral RNA Kit (TINGEN, Beijing, China) according to the manufacturer's protocols. Once the viral RNA was obtained, cDNA was generated in a 20 μl volume reaction mixture immediately using the PrimeScript™ RT reagent kit (TAKARA, Japan) according to the manufacturer's instruction. The preparation of VP1 fragments includes two amplification processes. First, a 50 μl reaction mixture containing 10 μl cDNA was used in the first round PCR using primers 222 and 224, and the reaction was under these conditions: 94°C for 5 min; 40 cycles at 94°C for the 30 s, annealing at 42°C for 30 s, and extension at 72°C for 1 min; and a final extension step at 72°C for 5 min. Then, 3 μl amplification product from the first round PCR was added to a second PCR with the primers AN88 and AN89 under the amplification conditions as described for the first round PCR but with annealing temperature of 60°C. The amplified products were purified from 1.2% agarose gels and subjected to sequencing using ABI PRISM310 Genetic Analyzer. Finally, the Basic Local Alignment Search Tool (www.ncbi.nlm.nih.gov/blast) was used to classify the types of enteroviruses.

### Phylogenetic Analysis

Sequence analysis of major serotypes of CVA6, CVA2, and CVA5 was performed using Molecular Evolutionary Genetics Analysis software (MEGA, version 6.0). Phylogenetic trees were built based on partial VP1 genes using the neighbor-joining method with MEGA 6.0 software. Validation of reconstructed evolution trees was supported statistically using the bootstrap with 1000 replicates.

### Statistical Analysis

Data were processed and analyzed using Microsoft Excel 2007 and IBM SPSS V.19.0 software (IBM Corp, Armonk, NY USA). All categorical variables were reported as frequency and percentage, and their differences among the groups were compared using the Pearson χ^2^ or Fisher's exact tests. Quantitative data were reported as median with interquartile range and were compared among the groups using the Kruskal–Wallis test. *P*-value < 0.05 was considered as statistically significant.

## Results

### Overall Distribution of EVs

**Figure 1:**
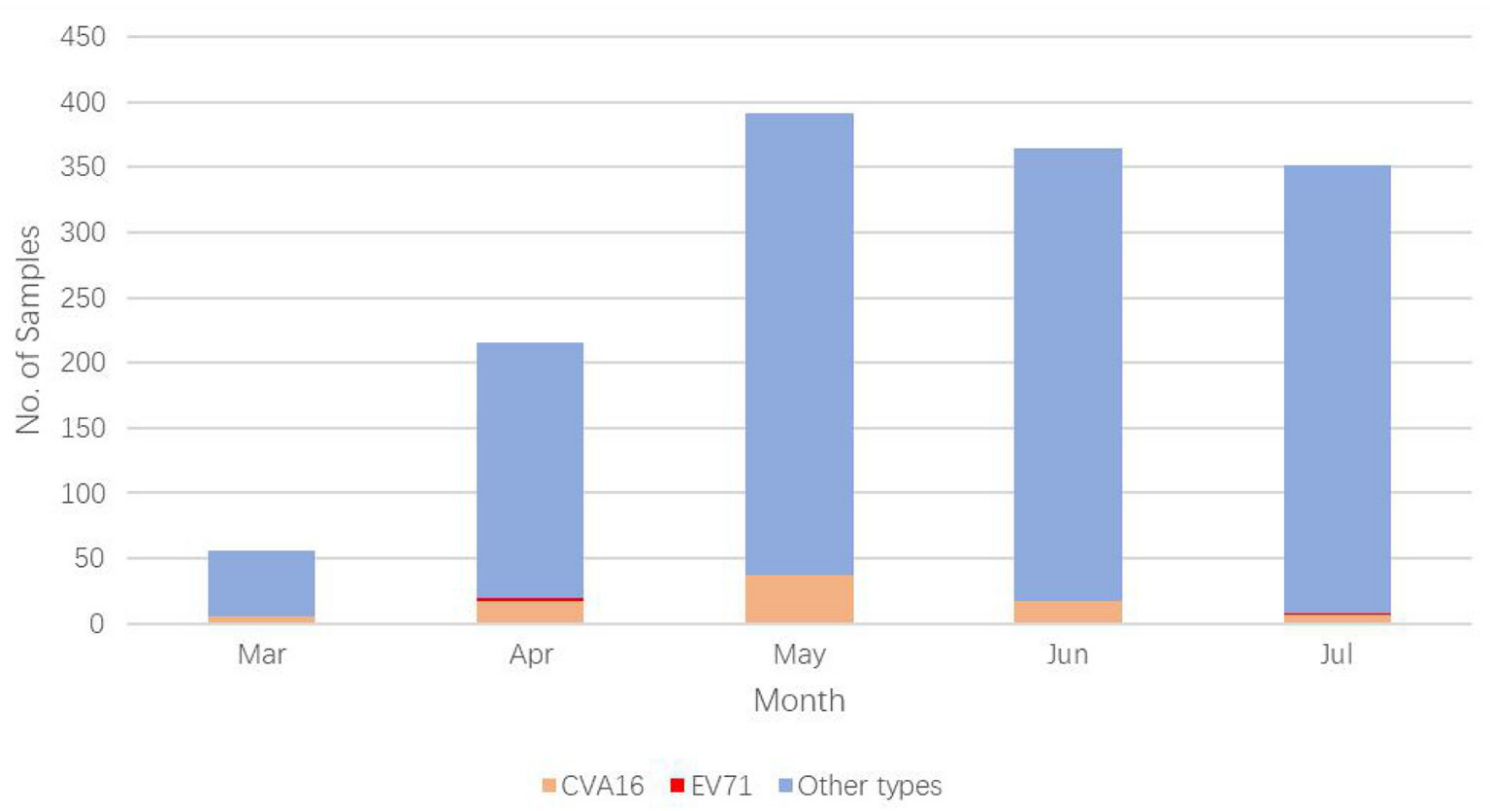
Distribution of EV types causing HFMD/HA in the year of 2019.

We have previously showed that a seasonal peak appears between March and July every year in Zunyi, China (15); we therefore conducted the epidemiological surveillance during the period. From 1 March to 31 July 2019, a total of 1,377 EVs associated HFMD/HA inpatient cases (823 HFMD and 554 HA cases) were enrolled. Of them, four were EV-A71 (0.3%, 4/1,377), 84 were CVA16 (6.1%, 84/1,377), and 1,289 cases (93.6%, 1,289/1,377) were associated with non-EV-A71/CVA16 infection (Figure 1).

### Typing of Non-EV-A71/CVA16 EVs

**Figure 2:**
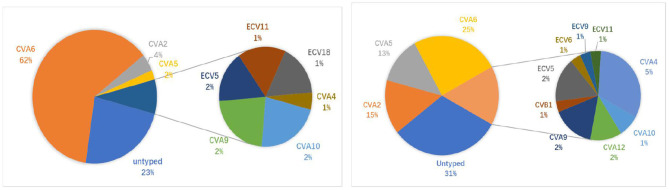
Percentage of non-EV-A71/CVA16 serotypes in HFMD/HA patients. **Left**: HFMD (166 cases in total); **Right**: HA (107 cases in total).

To identify the latest epidemic pathogen of HFMD/HA, 372 samples identified as types other than EV-A71/CVA16 cases were randomly selected and subjected to typing by partial VP1 gene sequencing. A total of 14 EV types have been successfully identified in 273 samples (73.4%), including 166 HFMD cases and 107 HA cases. Collectively, CVA6 (63.4%, 173/273), CVA2 (11.7%, 32/273), CVA5 (9.2%, 25/273) and CVA4 (3.3%, 9/273) were the most prevalent EV types associated with HFMD/HA cases (Figure 2). For HFMD cases, the three most common types were CVA6 (80.7%, 134/166), CVA2 (5.4%, 9/166) and CVA5 (3.0%, 5/166); similarly, for HA cases, the three most prevalent serotypes were CVA6 (36.5%, 39/107), CVA2 (21.5%, 23/107) and CVA5 (18.7%, 20/107). The proportion of CVA2, CVA4, and CVA5 were higher in HA cases than in HFMD cases, whereas the proportion of CVA6 was higher in HFMD cases than in HA cases (Table 1).

**Table 1:** The prevalence frequency of non-EV-A71/CVA16 EVs in HFMD/HA cases.

**EV types**	**HFMD (*n* = 166)**	**HA (*n* = 107)**	***P*-value**
	**No. of cases (%)**	**No. of cases (%)**	
CVA2	9 (5.4%)	23 (21.5%)	<**0.001**
CVA4	1 (0.6%)	8 (7.5%)	**0.003**
CVA5	5 (3.0%)	20 (18.7%)	<**0.001**
CVA6	134 (80.7%)	39 (36.4%)	<**0.001**
CVA9	4 (2.4%)	4 (3.7%)	0.716
CVA10	4 (2.4%)	2 (1.9%)	1
Others	9 (5.4%)	11 (10.3%)	0.125

*Bold values represents the values showing statistical significant difference*.

### Clinical Characteristics and Laboratory Results of Patients With Diverse Enterovirus Infections

To analyze the clinical features of EVs associated HFMD/HA, we included 84 patients who were infected with CVA16 as control at the same time. In all, clinical information and laboratory data were collected from 361 HFMD/HA cases. As shown in Table 2, there were 232 (64.3%) males and 129 (35.7%) females, with a male/female ratio of 1.8:1. The majority of patients were under 5 years old (*n* = 338, 93.6%), and about half of the patients were aged 1–3 years (54.6%, 197/361).

**Table 2:** Demographics of patients infected with different EVs on admission in this study.

**EV types**	**All**	**CVA6**	**CVA2**	**CVA5**	**CVA16**
No. of patients	*N* = 361	*N* = 173	*N* = 32	*N* = 25	*N* = 84
Males, no. (%)	232 (64.3%)	115 (66.5%)	23 (71.9%)	14 (56.0%)	51 (60.7%)
**Age groups (years)**					
≤ 1, no. (%)	87 (24.1%)	58 (33.5%)	6 (18.8%)	5 (20.0%)	10 (11.9%)
1–3, no. (%)	197 (54.6%)	92 (53.2%)	19 (59.4%)	11 (44.0%)	50 (59.5%)
3–5, no. (%)	54 (15.0%)	16 (9.2%)	5 (15.6%)	7 (28.0%)	20 (23.8%)
>5, no. (%)	23 (6.4%)	7 (4.0%)	2 (6.3%)	2 (8.0%)	4 (4.8%)

Clinical features of patients who were infected with CVA2, CVA5, CVA6 and CVA16 are shown in Table 3. Of the 361 cases, 60 (16.6%) were severe cases and 301 (83.4%) were mild HFMD/HA cases. The majority of the patients had initial clinical symptom of fever. Fever and fever higher than 39°C were significantly associated with enterovirus types. Compared to CVA16, CVA2, CVA5, and CVA6 were more likely to cause fever and high fever. In addition, compared with other EVs, a lower incidence of neurological complications was observed in CVA16 infected patients. In this study, all severe cases had neurological signs, including startled reaction (48.3%), twitching (31.7%), hand and foot trembling, and vomiting. Startled reaction was observed more frequently in patients who were infected CVA5 than CVA6. CVA5-infected cases more frequently developed severe HFMD/HA. Compared to CVA6, CVA5 and CVA2, CVA16 was less likely to cause severe HFMD/HA in this cohort. All patients were cured and discharged from the hospital.

**Table 3:** Clinical features of patients infected with different EVs on admission in this study.

**EV types**	**All**	**CVA6**	**CVA2**	**CVA5**	**CVA16**
No. of patients	*N* = 361	*N* = 173	*N* = 32	*N* = 25	*N* = 84
Fever (≥37.5°C)	327 (90.6%)	164 (94.8%)	31 (96.9%)	24 (96.0%)	64 (76.2%)^**#**^
High Fever (≥39°C)	205 (56.8%)	109 (63.0%)	27 (84.4%)	16 (64.0%)	27 (32.1%)^**#**^
Antiadoncus	145 (40.2%)	103 (59.5%)	21 (65.6%)	19 (76.0%)	54 (64.3%)
Neurologic complications	60 (16.6%)	33/173 (19.1%)	5/32 (15.6%)	6/25 (24.0%)	5/84 (6.0%)^**#**^
No. of patients	*N* = 60	*N* = 33	*N* = 5	*N* = 6	*N* = 5
Startle reaction	29 (48.3%)	22 (66.7%)	0 (0.0%)	2 (33.3%)^a^	5 (100.0%)
Twitching	19 (31.7%)	13 (39.4%)	4 (80.0%)	4 (66.7%)	0 (0.0%)
Hand and foot trembling	2 (3.3%)	1 (3.0%)	1 (20.0%)	0 (0.0%)	0 (0.0%)
Vomiting	3 (5.0%)	1 (3.0%)	1 (20.0%)	1 (16.7%)	0 (0.0%)
**Severe cases**	60 (16.6%)	33/173 (19.1%)	5/32 (15.6%)	6/25 (24.0%)	5/84 (6.0%)^**#**^

a *CVA5 vs. CVA16, P were < 0.05.*

# *Compared with CVA6, CVA2, and CVA5 groups, P were < 0.05*.

**Figure 3:**
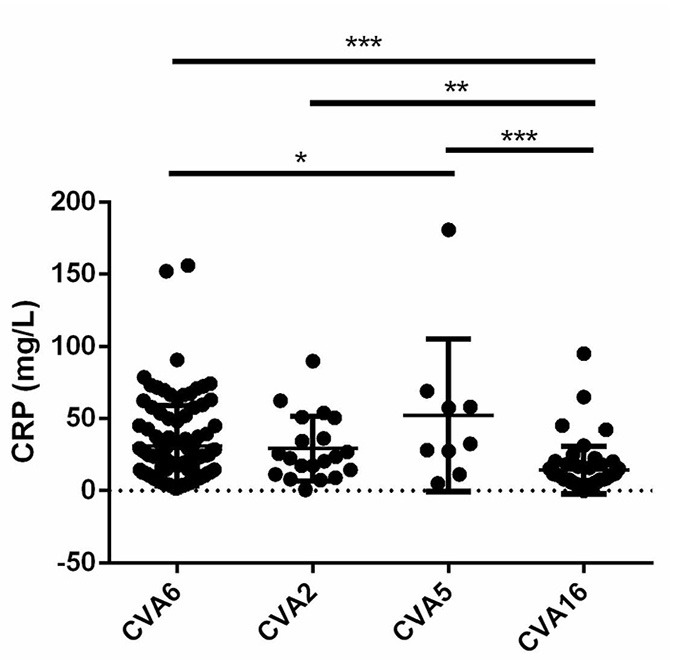
Levels of C-reactive protein (CRP) in cases with CVA6, CVA2, CVA5, and CVA16 (*P < 0.05; **P < 0.01; ***P < 0.001).

Laboratory data showed the neutrophil percentage was significantly higher in CVA5-associated cases than in CVA6- and CVA16-associated cases, and the lymphocyte percentage was correspondingly significantly lower in CVA5-associated cases than in CVA6- and CVA16-associated cases (Table 4). Elevated serum C-reactive protein (>10 mg/L) was more frequently observed in patients infected with CVA2 and CVA5 than patients infected with CVA16 (Table 4). Moreover, levels of CRP were higher than in patients infected with CVA5 than CV-A6 (Figure 3).

**Table 4:** Laboratory features of patients infected with different EVs on admission in this study.

**EV types**	**All**	**CVA6**	**CVA2**	**CVA5**	**CVA16**
No. of patients	*N* = 361	*N* = 173	*N* = 32	*N* = 25	*N* = 84
WBC (10^9^/L)	12.9 (9.9–16.6)	14.1 (10.4–17.7)	13.1 (11.7–16.0)	14.0 (12.2–16.4)	11.0 (9.1–14.8)
Neutrophil (%)	59.3 (46.7–70.3)	57.9 (46.1–70.1)	70.1 (50.2–77.0)	73.6 (60.9–81.5)^a^	55.0 (44.8–63.8)
Lymphocyte (%)	29.4 (19.6–39.2)	28.7 (20.5–41.0)	21.7 (13.8–35.9)	17.1 (11.8–26.8)^b^	32.2 (25.7–41.1)
Platelet (10^9^/L)	314.0 (252.0–375.0)	315.0 (256.0–383.0)	292.0 (246.3–357.0)	314.0 (243.0–345.0)	318.5 (268.8–371.5)
No. of patients	*N* = 359	*N* = 172	*N* = 32	*N* = 25	*N* = 84
AST (U/L)	38.7 (33.1–44.9)	39.2 (33.8–44.7)	38.9 (36.2–48.4)	39.1 (34.4–44.5)	37.6 (31.6–45.0)
ALT (U/L)	16.1 (13.1–20.2)	16.0 (13.6–20.7)	16.3 (13.6–19.9)	16.3 (12.4–20.3)	15.9 (12.6–18.7)
No. of patients	*N* = 212	*N* = 102	*N* = 20	*N* = 9	*N* = 52
CRP (mg/L)	19.8 (9.5–36.6)	23.3 (12.0–42.0)	23.2 (13.7–40.0)	32.5 (27.7–58.2)	9.9 (5.9–15.6)^**#**^
CRP (≥10 mg/L)	105 (49.5%)	53 (52.0%)	16 (80.0%)	8 (88.9%)	26 (50.0%)
No. of patients	*N* = 276	*N* = 137	*N* = 22	*N* = 19	*N* = 59
GLU (mmol/L)	5.0 (4.3–5.5)	5.0 (4.4–5.4)	4.6 (4.0–5.8)	5.5 (4.7–5.9)	4.9 (3.8–5.6)
GLU (≥6.1 mmol/L)	38 (13.8%)	15 (10.9%)	4 (18.2%)	3 (15.8%)	9 (15.3%)

a *CVA5 vs. CVA6 and CVA16, P were < 0.05;*

b *CVA5 vs. CVA6 and CVA16, P were < 0.05.*

# *Compared with CVA6, CVA2, and CVA5 groups, P were < 0.05.*

*AST, aspartate aminotransferase; ALT, alanine aminotransferase; CRP, C-reactive protein; GLU denotes glucose*.

### Phylogenetic Analyses

**Figure 4:**
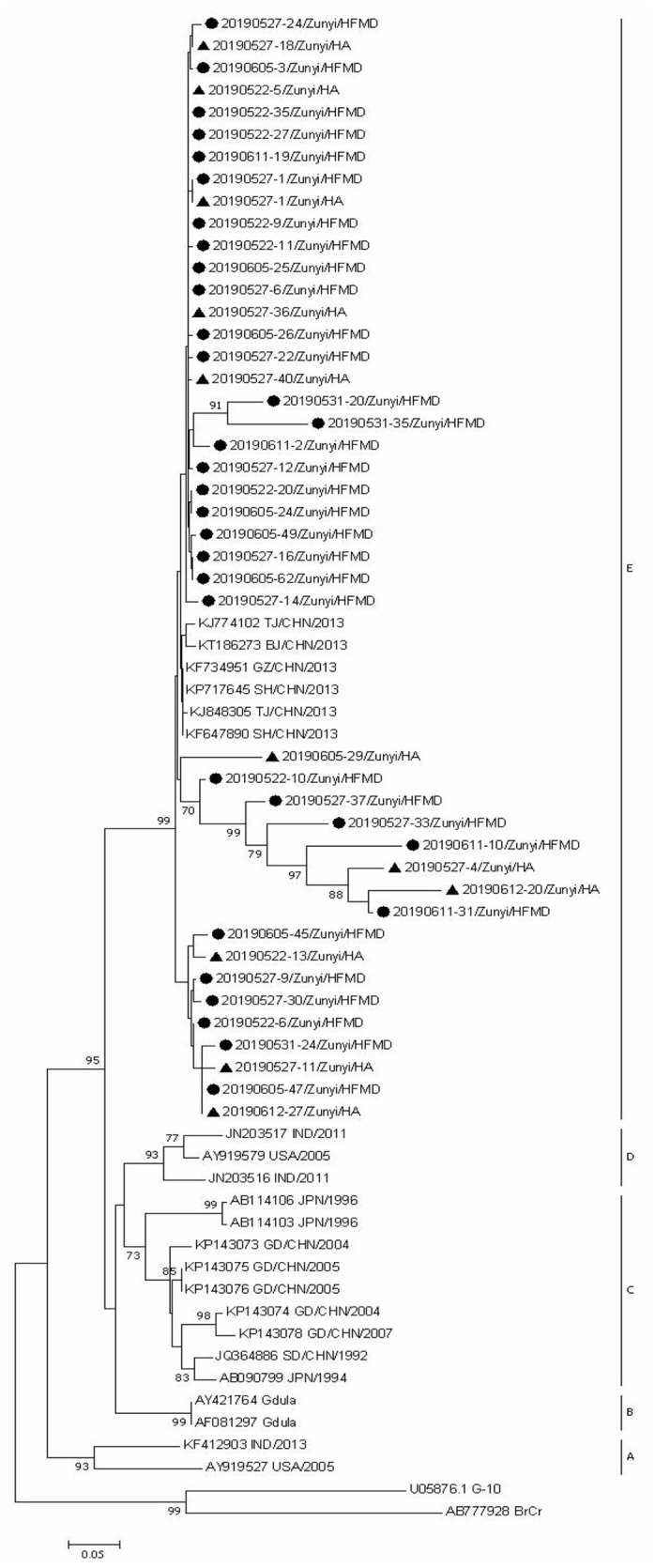
Phylogenetic analysis of CVA6 based on partial VP1 gene sequence. Strains identified from HFMD are marked by circle, and those identified from HA are marked by triangle.

**Figure 5:**
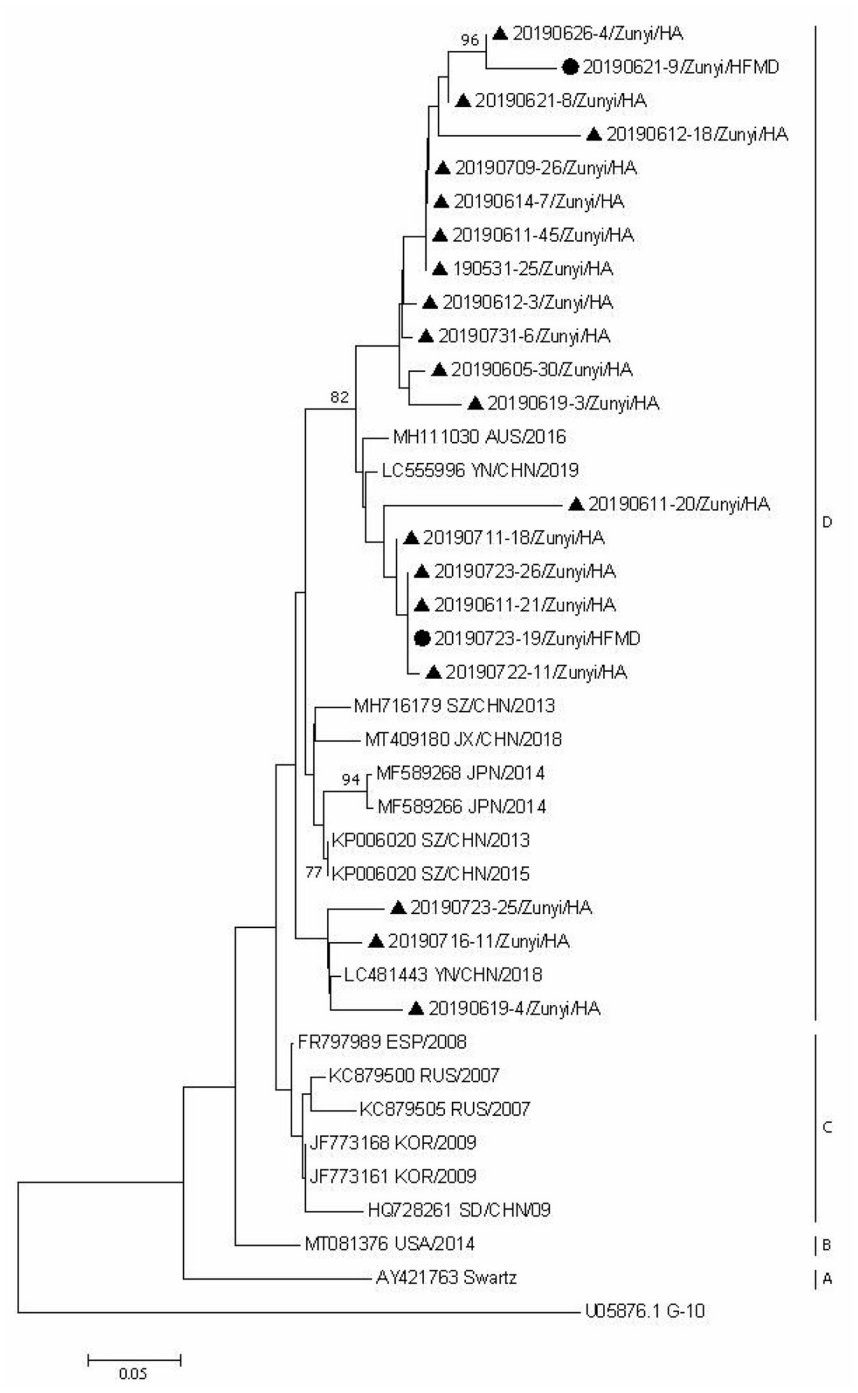
Phylogenetic analysis of CVA5 based on partial VP1 gene sequence. Strains identified from HFMD are marked by circle, and those identified from HA are marked by triangle.

**Figure 6:**
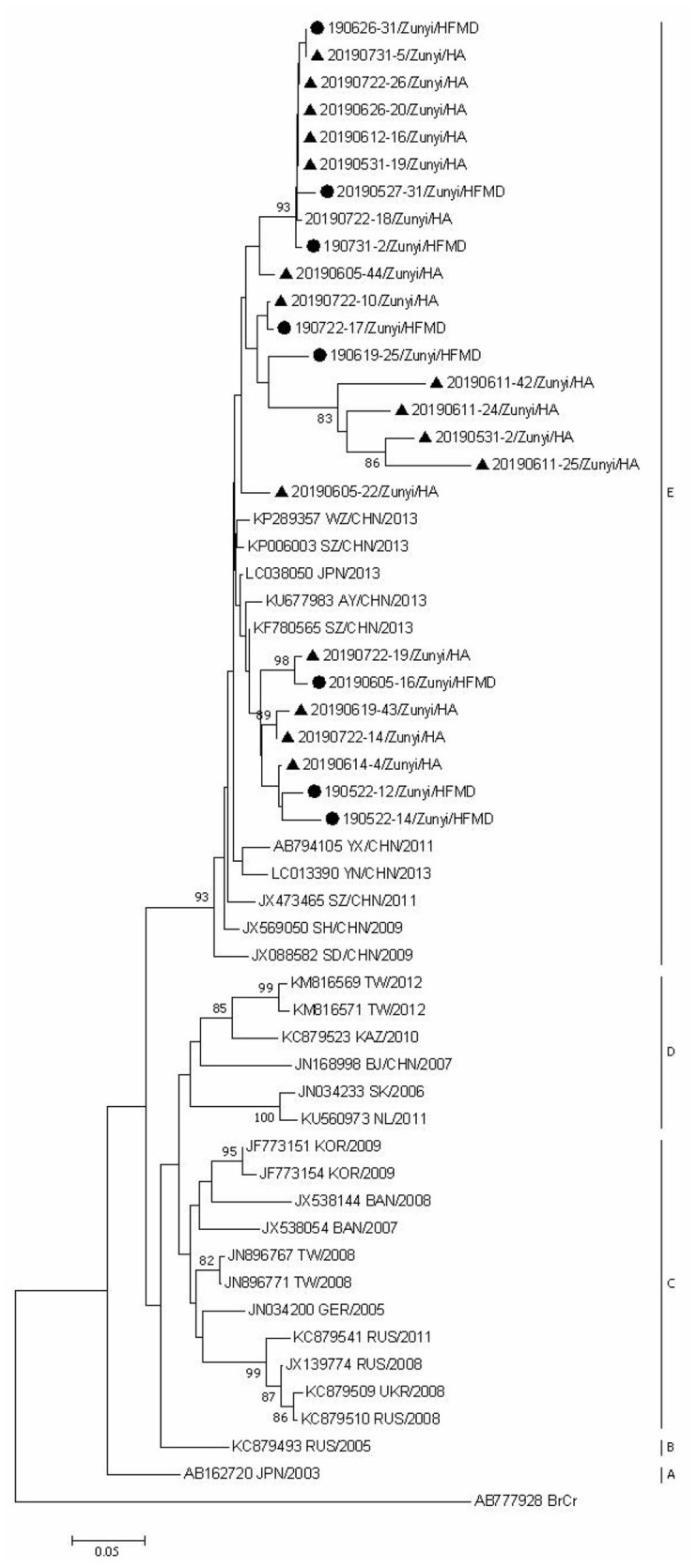
Phylogenetic analysis of CVA2 based on partial VP1 gene sequence. Strains identified from HFMD are marked by circle, and those identified from HA are marked by triangle.

Since CVA6, CVA2, and CVA5 were the main EV types circulating in Zunyi, we analyzed the evolutionary relatedness with their corresponding reference strains sequences available from GenBank. According to the phylogenetic relations, CVA6 evolutional dendrogram was separated into A–E. The nucleotide identities of CVA6 sequences in this study were 91–96.3%, and all CVA6 strains clustered in to subclade E. Phylogenetic analysis showed CVA6 circulating in Zunyi was in a close genetic relationship to the strains identified in Tianjin, Shanghai, Beijing, and Guangzhou, China (Figure 4). VP1 based evolutional dendrogram of CVA5 separated at the viruses phylogenetically into clades A-D. The present results showed that the nucleotide identities of CVA5 sequences in this study were 84.4–95.7%, and CVA5 strains belonged to subclade D, which were in a close genetic relationship to Chinese strains identified in other cities or provinces, such as Yunnan, Jiangxi, and Shengzhen, and they also showed high sequence identity to two strains from Japan and Australian (Figure 5). An evolutional dendrogram of CVA2 separated into A–E. In this study, the nucleotide identities of CVA2 sequences were 83.1–97.3%. Like CVA2 strains identified in other cities of China, CVA2 strains identified in Zunyi city segregated into subclade E (Figure 6).

## Discussion

Here we provide epidemiologic information of EV associated HFMD/HA in Zunyi, 2019. Although EV-A71 and CVA16 have long time been the main EV types associated with HFMD (19), it seems that CVA6 has replaced the two types and became the most predominant EV type associated with HFMD/HA in some regions in recent years (14, 20, 21). Our work provides important evidence for the requirement of further EV surveillance, diagnostic reagent and vaccine development.

In this study, in addition to CVA6, CVA2, and CVA5 were the most prevalent types causing HFMD/HA. Although they have occurred recently in Europe in 2016 and in the United States in 2001, few studies reported CVA2 and CVA5 as the main pathogens responsible for HFMD compared with EV-A71, CVA6, CVA10, and CVA4 (22–24). Given the high prevalence of CVA6, CVA2, and CVA5 in patients with HFMD/HA, sensitive and reliable typing tests for them may be crucial for daily clinical diagnosis.

Unlike HFMD, the hazards of HA disease may be underestimated because it isn't a notifiable disease in China and few studies analyzed the etiology of HA. The present study showed that CVA6 (36.4%), CVA2 (21.5%), and CVA5 (18.7%) were the most three prevalent types associated with HA. CVA10, CVA4, CVA5, and CVA2 have aslo been reported as the major types associated with HA in other regions, including France, Hangzhou, Korea, and Thailand (8, 23, 25, 26). Together these data suggest that HA outbreaks caused by CVA2 and CVA5 should be monitored in future. It is notable that only a very limited number of HFMD/HA cases were caused by EV-A71 in this study. Previous findings showed the number of HFMD caused by EV-A71 was reduced in Sichuan, China, which was thought to result from the implementation of the EV-A71 vaccination (27). In Zunyi, it remains unclear how much the EV-A71 vaccination has affected the local prevalence of EVs, since the EV-A71 vaccines were only available in March 2019, and few children were vaccinated with the vaccines in this region.

While CVA16-associated case is generally thought to self-limited, a fraction of patients also develops severe case (28–30). In agreement with previous findings, the majority of CVA16-associated cases presented with mild symptom and only a small number of cases were severe in this study. Of note, when compared to cases infected with other EV types, the incidence of fever caused by CVA16 was relatively lower and levels of white blood cell (WBC) and CRP were also lower.

To our knowledge, clinical features of CVA5-associated HFMD/HA cases were seldom reported. In the present study, it was found that, compared to CVA16, CVA5 was associated with higher incidence of severe cases, and patients with CVA5 infection seemed to have a higher WBC number and serum CRP level. Although all cases have rashes, the rashes caused by CVA5 were usually confined to one or two sites. Further multicenter studies are needed to confirm this finding.

It has been reported that CVA6 is associated with higher incidence of severe HFMD cases than other EV types and patients infected with CVA6 commonly present with high fever, onychomadesis, pigmentation, and vesicular rashes (31). In addition, it has been shown that CVA6 was more likely to cause atypical clinical symptoms than other EV types in children and adults (32). Indeed, we also observed onychomadesis and atypical vesicular rashes in some cases associated with CVA6 infection, however, this information was missing in the majority of the patients, and their differences were not able to analyze in the present study.

Phylogenetic analysis of CVA6, CVA5, and CVA2 strains showed that strains identified in this region were close to the corresponding strains from other regions of China, which suggest that the prevalent strains in this region were widespread circulating in China. The strains identified from HFMD and HA cases did not form distinctive clusters, suggesting that even the same EV type can cause different clinical signs and symptoms. Further studies are needed to determine the underlying mechanisms involved in the differences in clinical symptoms between HFMD and HA caused by the same EV types.

The present study has some limitations. First, the cases of HFMD included in this study were from one city. Second, some EV types were not determined because the partial VP1 fragment could not be amplified using the primers and some sequences were not determined by sequencing using the degenerate primers. Third, EVs co-infection with other viruses or bacteria was not determined which may influence accuracy on the description of clinical features and laboratory results for a specific EV type.

The results of our study indicate that CVA6, and CVA2 and CVA5 were the dominant serotypes causing HFMD/HA outbreak in Zunyi, China. Future studies in different regions are required to confirm the results of this study. This finding is of great importance as it strongly indicates that the currently routine clinical practice of identifying EV-A71 and CVA16 is not enough and diagnostic kits for CVA6, CVA5 and CVA2 are required. In addition, novel EV vaccines need to be able to cover the emerging new EV types.

## Data Availability Statement

The datasets presented in this study can be found in online repositories. The names of the repository/repositories and accession number(s) can be found below: Sequencing data was downloaded from the GenBank database (URL) under the accession numbers U05876, AB777928, KP143074, KP143078, KP143073, KP143075, KP143076, JQ364886, AB090799, AB114106, AB114103, AY421764, AF081297, KF412903, AY919527, JN203517, AY919579, JN203516, KF734951, KJ848305, KP717645, KF647890, KJ774102, KT186273, AY421763, MF589268, MF589266, KP006020, MH716179, MT409180, LC481443, KC879500, KC879505, JF773168, JF773161, HQ728261, MT081376, MH111030, LC555996, KP006020, FR797989, AB162720, KC879493, KC879509, KC879510, JX139774, KC879541, JN034200, JX538054, JX538144, JF773151, JF773154, JN896767, JN896771, KM816569, KM816571, KC879523, JN168998, JN034233, KU560973, KU677983, KF780565, LC038050, JX473465, KP289357, KP006003, AB794105, LC013390, JQ968964, JX088582, JX569050, AY421760.

## Ethics Statement

This study was in compliance with the Helsinki Declaration and was approved by the Human Research Ethics Committee of Zunyi Medical University Third Affiliated Hospital (The First People's Hospital of Zunyi). Informed consents were obtained from parents or guardians before sample collection.

## Author Contributions

WZ and KW: conceptualization and design. YA: writing. KW: funding acquisition, and supervision. YA, WZ, JW, JZ, MS, SY, CD, XL, DW, PT, XC, and HZ: experiments. YA, WZ, JZ, MS, SY, HZ, and JW: data collection and analysis. All authors contributed to the article and approved the submitted version.

## Conflict of Interest

The authors declare that the research was conducted in the absence of any commercial or financial relationships that could be construed as a potential conflict of interest.
